# Charge Regulation Enables Uptake of Ampholytes to Polyelectrolyte Brushes

**DOI:** 10.1021/acsmacrolett.5c00669

**Published:** 2025-12-29

**Authors:** Roman Staňo, Peter Košovan

**Affiliations:** † Department of Physical and Macromolecular Chemistry, Faculty of Science, Charles University, Hlavova 8, 128 40 Prague 2, Czech Republic; ‡ Yusuf Hamied Department of Chemistry, 2152 University of Cambridge, Lensfield Road, Cambridge CB2 1EW, United Kingdom

## Abstract

Uptake of proteins and ampholytic solutes into polyelectrolyte brushes underlies some biological processes and also applications in sensing or biomedicine. Especially uptake on the “wrong” side of the isoelectric point (pI) remains puzzling, with charge regulation and solute patchiness proposed as possible mechanisms. Using a hierarchy of approximations, coarse-grained molecular simulations, self-consistent mean-field, and a simple phenomenological model, we investigated the uptake of model ampholytic solutes into polyanionic brushes across varying pH, salt concentrations, p*K*_a_ values, and peptide sequences. In a narrow pH range on the wrong side of pI, charge regulation enables uptake of the ampholytes by inducing charge inversion so that they become positively charged in the brush despite being negatively charged in the bulk. This charge inversion can be calculated from the pH difference between the brush and the bulk, which is related to the Donnan potential. It is strongest for ampholytes with small differences between acidic and basic p*K*_a_ values and decreases with increasing salt. Our phenomenological model reproduces the universal effect of charge regulation promoting ampholyte uptake into brushes but fails to be quantitative. The mean field model is close to explicit simulations for alternating sequences, but fails to describe the effect of charge patchiness, which is only captured by explicit simulations. Thus, our phenomenological framework offers a practical rule of thumb for estimating uptake from experimentally accessible parameters without sophisticated calculations. Deviations from this rule of thumb for complex ampholytes, such as proteins or peptides with patterned charge sequences, are captured only by explicit simulations.

## Graphical abstract

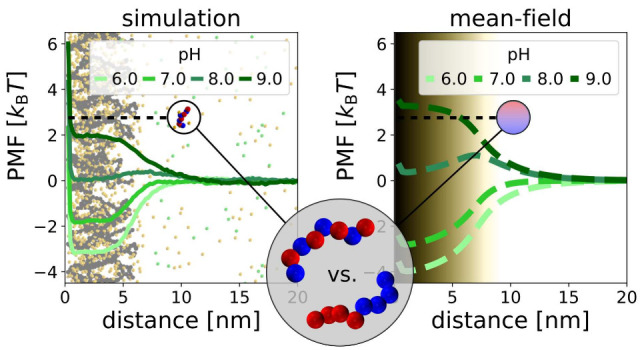

Uptake of proteins and other charged solutes into polymer brushes^1–3^ can be exploited for applications in sensors^4,5^ and biomedicine,^6,7^ but also plays a key role in cell division, where the *Ki*-67 peptide brushes coating chromosomes recruit RNA to drive interchromosome attraction.^8,9^ Upon uptake, the solute concentration inside the brush can be much higher than in the supernatant bulk, even by several orders of magnitude. In general, the uptake can be regulated by changing the salt conditions or the solution pH, which affects the ionization states of weakly acidic or basic groups, either on the polymer brush or on the solute molecule. Furthermore, these ionization states are affected by the local microenvironment in a process called charge-regulation,^10–12^ causing that charge of the solute inside the brush may significantly differ from its charge in the bulk solution at a given pH.

The pH-dependence of swelling and net charge of the polyelectrolyte brushes^13–24^ and the net charge of proteins and other ampholytic solutes in dilute solutions,^10,25–28^ respectively, can be considered well understood, while the description of their mutual interaction is a more complex problem. Strong attraction between proteins and polyelectrolytes has been observed in many cases when they are oppositely charged.^29–32^ For protein–polyanion interactions, this attraction trivially occurs below isoelectric point, i.e., at pH < pI, when the protein has a net positive charge. Conversely, for protein–polycation interactions, it occurs at pH > pI, when the protein has a net negative charge. In some cases, however, attraction has been observed also in the pH range when both polyelectrolyte and protein bear charges of the same sign, termed attraction on the “wrong” side of isoelectric point.^33,34^ This demonstrates that the role of electrostatics and charge regulation in protein–polyelectrolyte interactions is far from trivial and considering just the net charge of the protein in bulk solution is not sufficient. However, in contrast to the large amount of experimental work, theoretical studies of these interactions are much more limited.

When designing a molecular model of protein–polyelectrolyte brush interactions, simplifications are inevitable. Molecular simulations with all-atom resolution are not suitable for such problems because capturing the relevant length scales (tens of nanometers) and time scales (microseconds or more) is prohibitively expensive. On the other hand, coarse-grained models with reduced resolution have been successfully applied to study interactions of peptides with a single polyelectrolyte chain,^27,35^ a star^36^ or with a nanoparticle,^37–41^ identifying two main mechanisms driving the adsorption on the “wrong” side of the isoelectric point. First, if the solute contains a charged patch with a high charge density, it can adsorb onto the oppositely charged polymer, even if the net charge of the solute has the same sign as the polymer, as has been established for patchy nanoparticles interacting with polyelectrolytes in dilute solution.^38,42^ Second, mechanism is charge regulation on the solute. In response to the local environment determined by the polymer brush, the charge regulation can rescale or even invert the charge on the solute, provided that the pH is not too far from the isoelectric point,^12,36^ again as established for association in dilute solutions. Nevertheless, a polyelectrolyte brush has a different geometry, monomer density profile, and also the resultant electric field, as compared to single polyelectrolyte chains or stars with a low number of arms. Simulation studies of the uptake in brushes are rather scarce. The existing works either include the patchiness, but do not consider the charge regulation^43^ or consider the charge regulation, but do not consider the patchiness, and represent the whole protein as a single sphere with an ascribed net charge.^44^ Thereby, while both these studies observed the adsorption at the “wrong” side of the isoelectric point, they attributed it to different causes.

Similar contradictions arise also in the mean-field models of brushes, where the explicit point particles are replaced with the density fields minimizing the free energy of the system.^45,46^ Early mean-field studies suggested that the uptake should occur only if solute in the bulk solution has an opposite charge to the brush.^47^ Contrary to that, other mean-field studies predicted the uptake of such solutes also on the “wrong” side of the isoelectric point, using a generic model of ampholyte with acidic and basic groups^48–50^ or a model with p*K*_A_ values representing a specific protein.^51–53^ These mean-field models explained the observed effects in terms of the local electrostatic potential affecting the charge regulation of the model ampholyte or protein. Simultaneously, they emphasized the limitations of mean-field approximation in neglecting some correlations, which might be important, especially for more complex molecules with a nonuniform (patchy or patterned) distribution of ionizable sites. Apparently, a more systematic investigation of these phenomena with a unitary set of approximations consistent across the studied systems has not been conducted yet.

Herein, we provide a comprehensive study of the uptake of an ampholytic solute into a polymer brush, exploring the interplay of solution pH and peptide p*K*_A_, but also salt conditions and peptide sequence, outlining the conditions at which the adsorption takes place on the wrong side of pI. In contrast to the above simulation studies, we consider the charge regulation and patchiness simultaneously, directly analyzing their contributions to the uptake. To achieve this, we employ a hierarchy of three models at different levels of approximation, allowing us to systematically dissect the underlying physical chemistry: (1) particle based model for molecular simulations, (2) numerical self-consistent mean-field model and (3) analytically solvable phenomenological model.

The investigated system, to be represented with the three above models, is composed of a polyanionic brush and ampholytic octapeptide (*ab*)_4_ and later also *a*_4_ *b*_4_, where *a* and *b* represent an acidic and basic site with acidity constants p*K*_A_^acid^ and p*K*_A_^base^ respectively, as schematically shown in Figure 1. For convenience, we fix the isoelectric point to pI = 7 and define

$$\Delta pK=pK_{A}^{\mathrm{acid}}-pK_{A}^{\mathrm{base}}$$ 1

thereby p*K*_A_^acid^ = pI – Δp*K*/2 and p*K*_A_^base^ = pI + Δp*K*/2, similar to ref 25. In the current study, we used Δp*K* ∈ {0, 2, 4} to represent qualitatively different types of the titration behavior. The ideal titration curves of these three cases in Figure 1 show that at Δp*K* = 0, the ampholyte undergoes the strongest charge regulation at the isoelectric point because both acidic and basic residues change their ionization states in the same range of pH. In contrast, Δp*K* = 4 exhibits a weak charge regulation at the isoelectric point, whereas Δp*K* = 2 represents an intermediate case.

**Figure 1:**
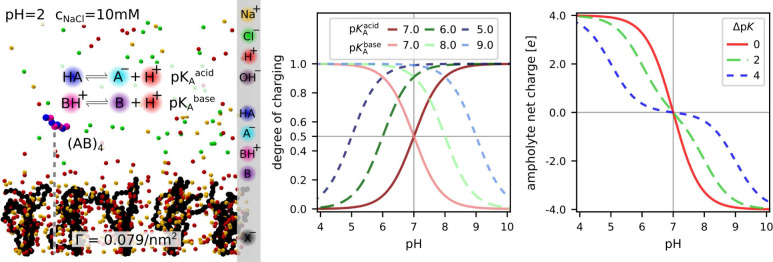
Left: schematic representation of the simulated system; Center: ideal titration curves of acidic and basic residues of the ampholyte; Right: total (ideal) net charge on the ampholyte;

First, the particle based model (1) utilizes the standard bead–spring model^54,55^ to represent both the brush and the ampholytic, solute as shown in Figure 1 and described in full detail in Section I in the SI. The brush is composed of negatively charged chains *N* = 25 monomers long, and grafted to a surface with density Γ = 0.079 chains/nm^2^. The bulk solution contains explicitly modeled monovalent ions (Na^+^, Cl^–^, H^+^, OH^–^), the concentrations of which determine the pH and ionic strength, the latter being fixed at *c* ^salt^ = 10 mM unless stated otherwise. We emphasize that the use of explicit ions is essential because their concentration significantly differs between the brush and the bulk. Correlations between the small ions, ampholyte and the brush determine the pH difference and also the extent of counterion release upon the uptake. Within the studied pH range, the salt concentration is approximately equal to ionic strength, therefore, we will use both these terms interchangeably in the discussion. The coarse-grained simulation model samples the configuration space using a combination of Langevin dynamics and Monte Carlo moves in the Grand-reaction ensemble.^56^ Therefore, it accounts for all interactions between all particles, especially intramolecular interactions and fluctuations of the ionization states on each site of the ampholyte. In the simulations, we used the weighted histogram analysis method (WHAM) to sample the interaction between the ampholyte and the brush. Its primary output is the potential of mean force (PMF) between the ampholyte and the brush, i.e., the free energy of interaction as a function of distance, which can be converted to the local concentration of the ampholyte, calculated as

$$c\left(r\right)=c^{\mathrm{bulk}}\exp\left(-\mathrm{PMF}\left(r\right)/k_{B}T\right)$$ 2

Second, the mean-field model (2) is based on the Scheutjens-Fleer self-consistent field theory, similar to refs 47, 57, and 58 , in detail explained in Section II in the SI. In contrast to the simulation model, the mean-field model assumes a density field, which is homogeneous and parallel to the grafting surface but can vary in the perpendicular direction. This model solves a set of propagator equations, which account for the connectivity of the chains, steric repulsion and electrostatic interactions on the Poisson–Boltzmann level. The primary output of these calculations are the concentration profiles, which can be converted to PMF using eq 2. Importantly, all particles in the mean-field model interact only with the surrounding density field. Therefore, the model neglects all intramolecular correlations and all density fluctuations parallel to the surface. All these correlations are included in the simulation model.

Finally, in the analytically solvable phenomenological model (3) we consider the polyelectrolyte brush as a two-phase system, where one phase is inside the brush and the other phase is the bulk solution far from the brush. It is a very crude approximation, because typically, the thickness of the brush–solution interface is comparable to the brush height. In reality, the local density gradually decreases inside the brush, followed by a steeper decrease at the brush–solution interface, and then it levels off to the bulk value far from the interface. If the brush–solution interface is comparable to the size of the whole brush, then the border between “inside” and “outside” is not uniquely defined. Consequently, the arbitrary choice of the border will affect the quantitative results but should not affect the qualitative picture. On the contrary, in sufficiently high brushes composed of long chains or in bulk gels immersed in solution, the interfacial region is negligibly small, so its precise location is unimportant. Despite some crude simplifications, the two-phase approximation allows us to conceptualize the uptake using relatively simple terms and formulas. Within this approximation, the uptake can be characterized by the distribution ratio of species *i* of valency *z*_*i*_, which follows the Boltzmann distribution in the electrostatic potential (Donnan potential) that builds up across the brush-solution interface

$$D_{i}=\frac{c_{i}^{\mathrm{brush}}}{c_{i}^{\mathrm{bulk}}}=\exp\left(\frac{z_{i}^{\mathrm{brush}}\psi^{\mathrm{brush}}-z_{i}^{\mathrm{bulk}}\psi^{\mathrm{bulk}}}{k_{B}T}\right)=e^{z_{i}^{\mathrm{brush}}\psi_{\mathrm{Don}}/k_{B}T}$$ 3

where *c* is concentration and ψ is the local electrostatic potential, ψ_Don_ = ψ^brush^ – ψ^bulk^ and *k*_B_ *T* is the thermal energy. If species *i* is charge-regulating, then its valency inside the brush, *z*_*i*_^brush^, will generally differ from the bulk value, *z*_*i*_^bulk^. By choosing the boundary condition ψ^bulk^ = 0, we eliminate *z*_*i*_^bulk^, noting that only valency inside the brush is relevant for the uptake. Within the Donnan approximation, the distribution ratio of the monovalent ions, and hence, the Donnan potential can be calculated as^56^

$$D_{\pm }=e^{\pm \psi_{\mathrm{Don}}/k_{B}T}=\frac{c_{A^{-}}}{2I}\pm \sqrt{{\left(\frac{c_{A^{-}}}{2I}\right)}^{2}+1},\left(\mathrm{ideal}\mathrm{Donnan}\right)$$ 4

where *c*_A^–^_ is the concentration of ionized acidic groups on the polymers inside the brush and *I* is the ionic strength of the bulk solution. Specifically for H^+^ ions, we can define the quantity

$$\delta \equiv -\log_{10}D_{H^{+}}=\frac{e^{\psi_{\mathrm{Don}}/k_{B}T}}{\ln\left(10\right)}$$ 5

related to the local concentration of H^+^ ions inside the brush. In the simplified two-phase model, it could be interpreted as the effective pH inside the brush: pH_eff_ = pH + δ. As discussed elsewhere, this equation can be equivalently interpreted so that the local interactions shift the p*K*_A_ of each ionizable group to p*K*_eff_ = p*K*_A_ – δ.^59^ Neglecting all other interactions, the degree of ionization of each acid and base group on the ampholyte inside the brush can be calculated by using a modified Henderson–Hasselbalch equation:

$$\mathrm{pH}+\delta -pK_{A}=-\log_{10}\frac{\alpha }{1-\alpha }$$ 6

The net charge of the ampholyte inside the brush then follows as

$$z_{\mathrm{amp}}^{\mathrm{brush}}=\underset{j}{\sum }z_{j}\alpha_{j}\left(\mathrm{pH}+\delta \right)$$ 7

where we explicitly indicate that the ionization degrees α_*j*_ depend on pH and δ. The summation runs over all ionizable groups on the ampholyte and *z*_*j*_ = ±1 for the base and acid, respectively. The distribution ratio of such ampholyte then becomes

$$D_{z}=10^{-z_{\mathrm{amp}}^{\mathrm{brush}}\delta }$$ 8

Thus, the full procedure of estimating the uptake is as follows: (1) Estimate *c*_A^–^_ from the grafting density and height of the brush; (2) Calculate the Donnan potential using eq 4 and δ using eq 5; (3) Calculate *z* ^brush^ using eqs 6 and 7; (4) Calculate the distribution ratio using eq 8. Ultimately, this simple model allows us to estimate the distribution ratio of the ampholyte based on a minimal set of parameters: concentration of acidic groups inside the brush, pH and ionic strength of the bulk solution, and ampholyte composition (number and p*K*_A_ values of its ionizable groups). All these parameters are experimentally accessible, or can be estimated from experimentally determined quantities. This phenomenological model neglects all nonelectrostatic interactions and accounts for the p*K*_A_ values of various ionizable sites on the solute but it does not account for their distribution.

First, let us consider a simple ampholyte (*ab*)_4_, composed of 4 weakly acidic and 4 weakly basic monomers, arranged in an alternating sequence, with p*K*_A_^acid^ = p*K*_A_^base^ = pI = 7, i.e., Δp*K* = 0. The top panels of Figure 2 show the potential of mean force experienced by this ampholyte at various distances from the brush and at various pH values. For convenience, the right axis of these panels shows the corresponding local concentration. The bottom panels of Figure 2 show the net charge of this ampholyte under the same conditions. Qualitatively, the results from self-consistent field calculations (left column) and coarse-grained simulations (right column) look similar. Therefore, in the following discussion, we will first address the common features observed in both data sets, and then we focus on the differences.

**Figure 2:**
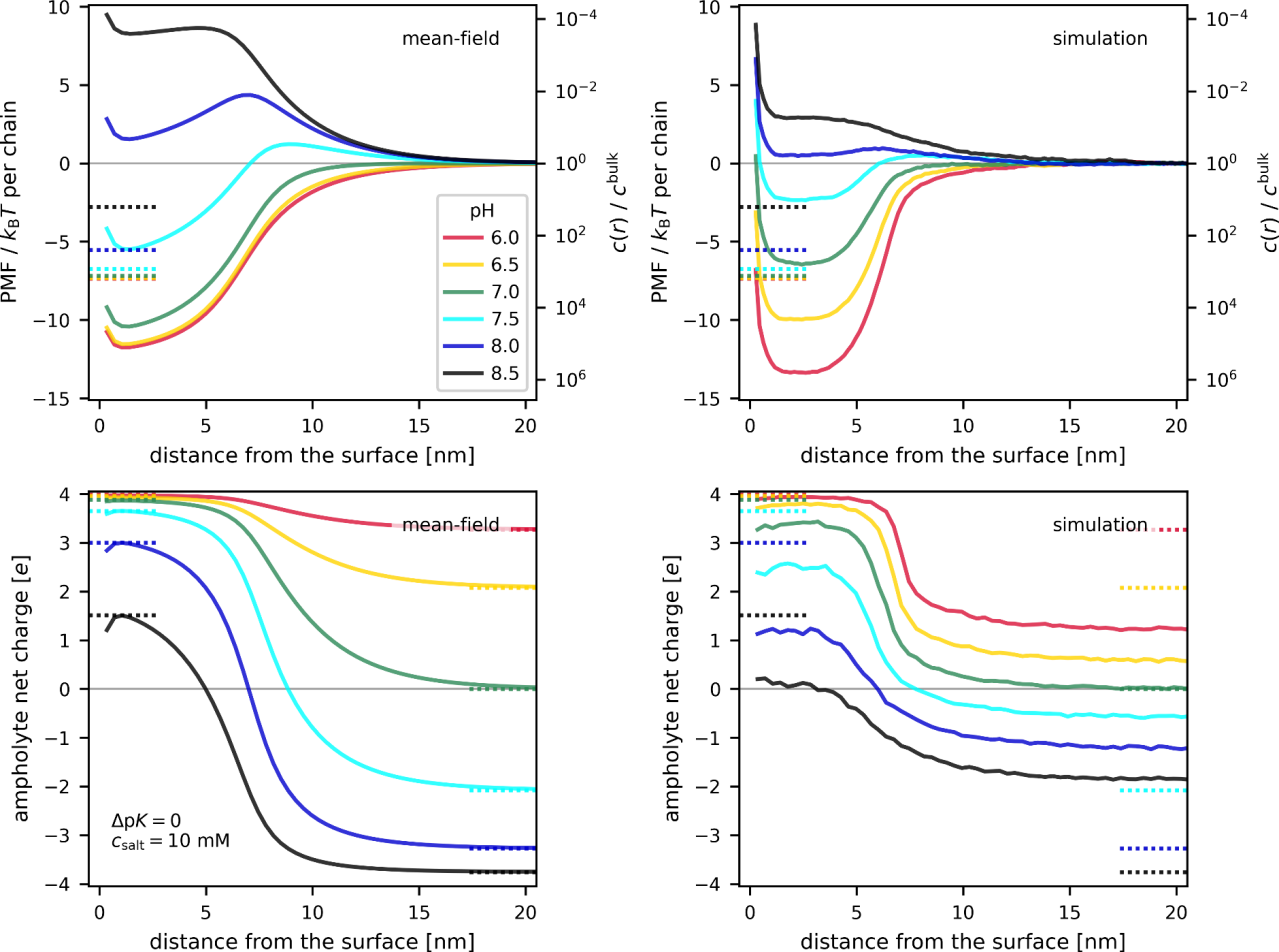
Uptake of an (*ab*)_4_ ampholyte with p*K*_A_^acid^ = p*K*_A_^base^ = 7 into anionic polyelectrolyte brush at various pH values. Dotted horizontal lines are the solutions of the two-state phenomenological model. Top row: potentials of mean force (left axis) and local concentration profile (right axis) as a function of distance from the surface; Bottom row: Net charge on the ampholyte as a function of distance from the surface; Left column: results of mean-field calculations; Right column: results of coarse-grained simulations.

At pH < 7 = pI, this ampholyte is positively charged in the bulk. Accordingly, the potential of mean force exhibits a pronounced minimum at short distances (inside the brush), indicating uptake of the ampholyte. Under these conditions, net charge of the ampholyte is positive in the bulk but becomes more positive as it enters the brush, making the interaction with the brush more favorable. Thus, at pH < 7 = pI the uptake occurs trivially on the “right” side of the isoelectric point, but it is enhanced by charge regulation. As the pH is increased, the minimum in the PMF gradually decreases and simultaneously a local maximum appears at intermediate distances, where the brush-solution interface is located. Concomitantly, net charge of the ampholyte in the bulk approaches zero and eventually becomes negative at pH < pI. Interestingly, the minimum in PMF at short distances, indicating uptake of the ampholyte, persists up to pH = 8 > pI. At 7 < pH < 8.5, the ampholyte is negatively charged in the bulk but positively charged inside the brush, indicating that uptake of the ampholyte into the brush could be enabled by charge regulation. Up to pH = 7.5, the depth of the minimum of PMF inside the brush gradually shrinks but it remains global minimum, indicating uptake of the ampholyte into the brush. At pH = 8 it becomes a local minimum, separated by a free energy barrier from the global minimum which is found at an infinite distance from the brush. Then, the ampholyte is expelled from the brush-solution interface while its concentration deep inside the brush is only slightly lower than in the bulk. Eventually, at pH = 8.5, the local minimum practically vanishes and PMF inside the brush attains a higher positive value, corresponding to expulsion of the ampholyte not only from the interface but also from the entire brush. In the pH range 7.0–8.0, the local maximum in PMF at the brush–solution interface poses an additional kinetic barrier, potentially preventing the ampholyte from entering the brush. However, if the height of this barrier is just a few *k*_B_ *T*, it can be easily crossed; therefore, the ampholyte can accumulate inside the brush also on the “wrong side” of the isoelectric point, i.e., under conditions when it is negatively charged in the bulk solution. Finally, as shown in Figure S3 in the SI, the same qualitative description applies also to the shortest possible ampholyte: dimer *ab*. The main difference is that the minimum in the PMF is much smaller for the shorter ampholyte (up to −4*k*_B_ *T*), resulting in a much weaker uptake. Interestingly, within the mean-field picture, the uptake on the wrong side of the isoelectric point occurs within the same pH range for both short and long ampholyte. Furthermore, our results on the *ab* dimer can be directly compared with the simulations in ref 44. Qualitatively, we observe the same trends, but quantitatively, the results slightly differ because they used a single-bead model of the ampholyte, yielding a higher absolute charge in the bulk at the same pH.

Both mean-field and simulation results in Figure 2 equally support the qualitative picture from our phenomenological model, but they differ in some important details. In the bulk, the mean-field predicts that net charge of the ampholyte follows the ideal HH equation, whereas net charge obtained from the simulations is closer to zero due to intrachain correlations.^58^ On the other hand, ampholyte charge inside the brush is much more consistent between the mean-field and simulations, because it is dominated by interactions of the ampholyte with the field of the brush. Furthermore, the mean-field predicts a slightly deeper minimum in PMF at high pH values and a gradual change of PMF as a function of distance. In contrast, the simulations predict a rather flat PMF profile inside the brush, followed by a sharper transition at the interface. Nevertheless, both of these profiles are consistent with the phenomenological picture of charge-regulation as a response to the local pH difference between the bulk solution and interior of the brush. The differences between the mean-field and simulation picture become more significant close to the isoelectric point, where the PMF inside the brush is close to zero. At pH = 7, the mean-field model predicts a more pronounced minimum in the PMF, suggesting a stronger uptake than in the simulations. At pH = 7.5, the mean-field model predicts a significant maximum in the PMF at the brush-solution interface and a local minimum inside the brush, indicating expulsion of the ampholyte, whereas the simulation yields a rather flat PMF profile, meaning that the ampholyte concentration is approximately constant throughout the system. Close to the isoelectric point, the uptake or expulsion of the ampholyte is a result of a subtle balance of competing effects, including steric effects and charge–charge correlations. In this respect, the simulation model is more accurate because it accounts for local correlations beyond the mean-field approximation. In principle, the mean-field model could be augmented by accounting for intramolecular correlations within the solute, for example, using the rotational isomeric state model,^60^ but in our opinion such an extension would not change the qualitative picture. In the case of Figure 2, the differences between the mean-field and simulations can be viewed only as small perturbations of the general trend. Later, we will show that in some other cases the different treatment of correlations in the two models leads to qualitatively different predictions.

Figure S6 shows the PMF profiles for ampholytes of various lengths, ranging from *N* = 2 (diblock (*ab*)_1_) up to *N* = 124. It shows that the depth of the PMF at pH = 8 increases as *N* is increased, but it reaches a saturation value at PMF ≈ −12*k*_B_ *T* per chain, which remains unchanged for *N* ≥ 8. When plotted as PMF per monomer, it is approximately constant for short chains, indicating that the contribution of various monomers is approximately additive. Beyond the saturation value, the steric effects due to the chain connectivity compete with the electrostatic effects, causing that the PMF per chain remains constant. At pH = 7 the PMF per chain scales linearly with *N* for short chains, but increases less steeply for *N* ≥ 32. Accordingly, the PMF per monomer remains constant up to *N* = 32 but then starts decreasing. This suggests that there is an upper limit to the height of the kinetic barrier at the interface so that even long chains should be able to cross it with non-negligible probability.

Finally, the dotted horizontal lines in Figure 2 represent the simple phenomenological model, where we used the average end-to-end distance from CG simulations as a proxy for brush height. The net charge inside the brush from the simple model very well agrees with the mean-field results whereas it agrees much less with the CG simulation results. In spite of good agreement with the net charge, the potential of mean force inside the brush from the simple model does not very well match the mean-field results. At pH < pI, the simple model underestimates the uptake (PMF), whereas at pH ≳ pI it overestimates the uptake, predicting that it should occur even at pH = 8.5. Even stronger disagreement with PMF from simulations is not surprising, since the simple model already failed to quantitatively capture the net charge. Nevertheless, the simple model succeeded in predicting the key trends with respect to pH: strong uptake at pH < pI, gradually vanishing uptake within a certain range on the “wrong” side of the isoelectric point, and explusion from the brush only at pH values sufficiently higher than pI. Thus, in general, the uptake of ampholytes into the brush is reasonably described by the phenomenological picture, suggesting that the Donnan potential at the brush solution interface can trigger a charge inversion of the ampholytic solute, resulting in its uptake even on the “wrong” side of the isoelectric point.

In Figure 3 we examine how the uptake of the ampholytes at a given pH is affected by the value of Δp*K*. For simplicity, we focus only on three pH values around the isoelectric point, pH ∈ {6, 7, 8} and three values of Δp*K* ∈ {0, 2, 4}. At pH = 6, all ampholytes are positively charged in the bulk. The ones with a smaller Δp*K* are more positive but still quite far from the maximum possible charge. Inside the brush, the ones with Δp*K* = 0 attain the maximum positive charge inside the brush due to charge regulation, closely followed by the ones with Δp*K* = 2. Only the ampholytes with Δp*K* = 4 attain a charge, which is well below the maximum. Consequently, we observe an uptake that is significantly enhanced by charge regulation. This effect decreases as the Δp*K* value is increased, resulting in a weaker uptake. Nonetheless, in all cases, this uptake is stronger with charge regulation than without.

**Figure 3:**
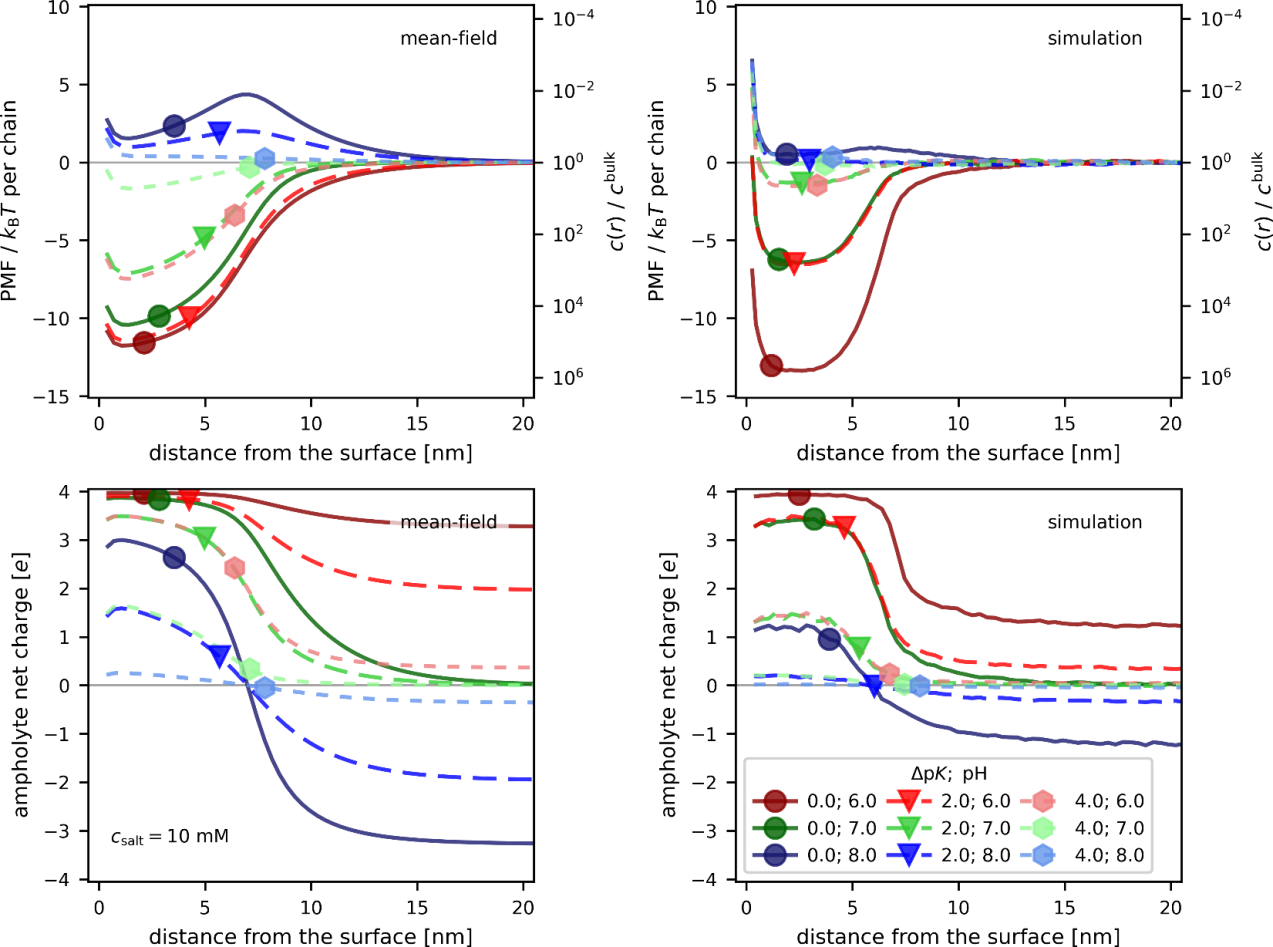
Uptake of (*ab*)_4_ ampholytes with various Δp*K* values at selected pH values. Top row: potentials of mean force (left axis) and local concentration profile (right axis) as a function of distance from the surface; Bottom row: Net charge on the ampholyte as a function of distance from the surface; Left column: results of mean-field calculations; Right column: results of coarse-grained simulations. Note that some results practically coincide, causing various types of points to appear on the same line.

At pH = 7.0, all ampholytes, irrespective of Δp*K*, are net neutral in the bulk. This implies that within each ampholyte the ionization degrees of acidic and basic groups are the same. Nevertheless, they differ between ampholytes with different Δp*K*, as well as between simulations and mean-field calculations. Accordingly, Figure 3 shows that they experience different extents of charge regulation as they enter the brush. The ampholyte with Δp*K* = 0 becomes most positively charged inside the brush, resulting in an uptake that is comparable to that at pH = 6.0 according to the mean-field model and somewhat weaker uptake according to the simulations. Finally, the most interesting case is the “wrong” side of isoelectric point, pH = 8.0, when the ampholytes are negatively charged in the bulk. The ampholyte with Δp*K* = 0 has the most negative charge in the bulk, but it also undergoes the strongest charge regulation as it enters the brush. As a result of this interplay between uptake and charge regulation, Figure 3 shows that at pH = 8 all these ampholytes have rather flat PMF profiles, indicating weak expulsion from the brush based on the mean-field model, and no expulsion at all based on the simulations. In summary, Figure 3 shows that the strongest uptake triggered by charge regulation occurs for ampholytes with a small Δp*K*. Simultaneously, the ampholytes with a small Δp*K* attain more positive or negative charge as the pH deviates from the isoelectric point. Their uptake at and below the isoelectric point is enhanced by charge regulation. Above the isoelectric point, the uptake is a result of subtle balance between charge regulation and other interactions, causing that the ampholyte may be weakly expelled from the brush, even if it can undergo charge inversion upon crossing the brush-solution interface. This observation is well captured by both mean-field and simulations but it is not captured by the simple phenomenological model which only considers charge regulation.

To further establish the relation between the charge regulation, Donnan potential, and the uptake, in Figure 4 we plot the distribution ratio as a function of pH at one selected salt concentration and different Δp*K* values (left panel) and different salt concentrations at one selected Δp*K* (right panel). These distribution ratios directly link our model predictions to experimental isotherms, which typically determine the adsorbed amount of solute as a function of the solute concentration under given conditions (pH, *c* ^salt^). The distribution ratio determines the slope of these isotherms in the linear regime at low solute concentrations. For example, the left panel of Figure 4 shows that the distribution ratio descreases as the salt concentration is increased. The same trend is observed in the initial slope of experimental isotherms in Figure 6 of ref 33, which studied the adsorption of bovine serum albumin in poly­(acrylic acid) brushes. Similarly, the right panel of our Figure 4 shows that the distribution ratio decreases as the pH is increased at fixed *c* ^salt^, in accordance with the initial slope of isotherms in Figure 8 of ref 33. Having established the connection to experimental isotherms, below we discuss and explain how the observed distribution ratios (adsorbed amount) should depend on the pH and *c* ^salt^ in the limit of low solute concentration.

**Figure 4:**
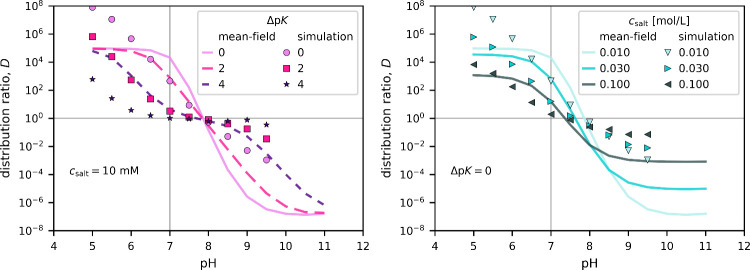
Distribution ratios of (*ab*)_4_ ampholytes as a function of pH under various conditions, comparing mean-field results (lines) to explicit-particle simulations (symbols). Left panel: various values of Δp*K* at fixed *c* ^salt^ = 10 mM. Right panel: various values of *c* ^salt^ at fixed Δp*K* = 0.

If the ionic strength (salt concentration) is much smaller than the concentration of ionized groups inside the brush, the distribution ratio from eq 4 and δ from eq 5 can be approximated as

$$D_{\pm }\approx \frac{c_{A^{-}}}{I},\delta \approx -\log_{10}\frac{c_{A^{-}}}{I},\mathrm{for},c_{A^{-}}\gg I$$ 9

This approximation breaks down at high salt, *c* ^salt^ ≈ 1 M, when the salt concentration becomes comparable to the concentration of ionized groups inside the brush (Figure S2 in the SI). Accordingly, when keeping the salt concentration fixed, the value of δ is fixed and the value of Δp*K* determines under which conditions the charge regulation enables the uptake. Indeed, the left panel of Figure 4 shows that in such a case, the dependence *D*(pH) qualitatively follows the trend of the net charge as a function of pH in Figure 1. Interestingly, all curves for various Δp*K* from both mean-field and simulations seem to cross at the same point: *D* = 1 and pH = 8, one unit of pH beyond the isoelectric point, pI = 7. This intersection is an approximate center of symmetry of the whole figure. Therefore, we discuss only its left half, noting that all arguments apply equivalently to the other half. Between pH = 7 and 8, we observe that the uptake on the wrong side of the isoelectric point significantly differs, depending on Δp*K*. At Δp*K* = 0, the distribution ratio steeply increases as the pH is increased. This increase is much less steep at Δp*K* = 2 and very weak at Δp*K* = 4, again following the trend of the net charge as a function of pH in Figure 1. At sufficiently low pH, the uptake predicted by the mean-field saturates at *D* ≈ 5 × 10^4^. This saturation is reached much earlier for smaller Δp*K* values. The simulations always show a slower increase in *D*(pH), which roughly corresponds to the mean-field results with (Δp*K* – 2). Furthermore, the simulations predict a much higher saturation value of *D* ≳ 10^8^, which appears out of scale of our current plot but can be estimated from the known dependence of the net charge on pH, which determines the charge regulation (Figure 1).

In accordance with the approximate equation in eq 9, the right panel of Figure 4 shows that an increase in the salt concentration at fixed Δp*K*_A_ decreases the saturation value of the distribution ratio. Simultaneously, the curves become less steep and then no longer cross at the same point. Still, we observe an uptake on the wrong side of the isoelectric point, however, the extent of the uptake decreases while simultaneously the pH window of this effect shrinks. Eventually, at Δp*K* = 4 and *c* ^salt^ ≈ 0.1 M, the effect practically vanishes, yielding *D* ≈ 1 at the isoelectric point. This general trend is reproduced by both mean-field and simulation results, although they differ quantitatively in the saturation values of *D* and in the pH window, where we observe the uptake. This effect of salt can be fully rationalized based on the phenomenological description: (1) the Donnan potential decreases, resulting in a lower distribution ratio at a given charge (cf. eq 9); (2) simultaneously, the value of δ decreases, suppressing the charge regulation. Finally, we note that the apparent similarity between the simulation results and mean-field at different Δp*K* values is just a numerical coincidence. Such a coincidence is not observed for a diblock ampholyte, as shown in Figure S5.

Finally, in Figure 5 we demonstrate that the effect of charge patchiness cannot be captured by the mean-field model and by the phenomenological model because it goes beyond the mean-field approximation. In this figure, we compare an alternating sequence (*ab*)_4_ with a block-like (patchy) sequence *a*_4_ *b*_4_ at pH = 8 and Δp*K* ∈ {0, 2, 4}. Within the mean-field approximation, the net charge on the ampholyte as a function of its location is unaffected by the sequence. The corresponding potentials of mean force and density profile are also almost identical for both sequences, suggesting that the ampholyte should be weakly expelled from the brush. Tiny differences between the two sequences occur only at the brush-solution interface. They can be ascribed to the dipolar nature of the block-like sequence, resulting in its preferential orientation in the interfacial region with a strong gradient of the local electrostatic potential. In contrast, the simulation results show a significant impact of the sequence on both charge regulation and uptake. The simulation results for the alternating sequence reasonably correspond to the mean-field. However, the results for patchy sequences indicate significant uptake for all Δp*K* values. At Δp*K* = 4 the charge regulation is negligible, nevertheless, we observe an uptake due to charge patchiness, similar to earlier studies.^36,43^ At Δp*K* = 0, charge regulation is significant, which further enhances the uptake to a level comparable to the maximum value observed for alternating sequences. This difference between differently patterned sequences is completely missed by all mean-field models but can be well captured by molecular simulations with explicit particles. This underpins the need to explicitly consider both charge regulation and charge patterning if we want to quantitatively describe the uptake of complex solutes, such as longer peptides or proteins. The key difference is that real peptides and proteins possess complex and irregular distributions of ionizable groups, more than two different p*K*_A_ values, as well as secondary and tertiary structure constrains which limit their conformational flexibility. These features are unique for each protein; nevertheless, their adsorption to polyelectrolyte gels and brushes should follow the same physical principles as outlined in our study.

**Figure 5:**
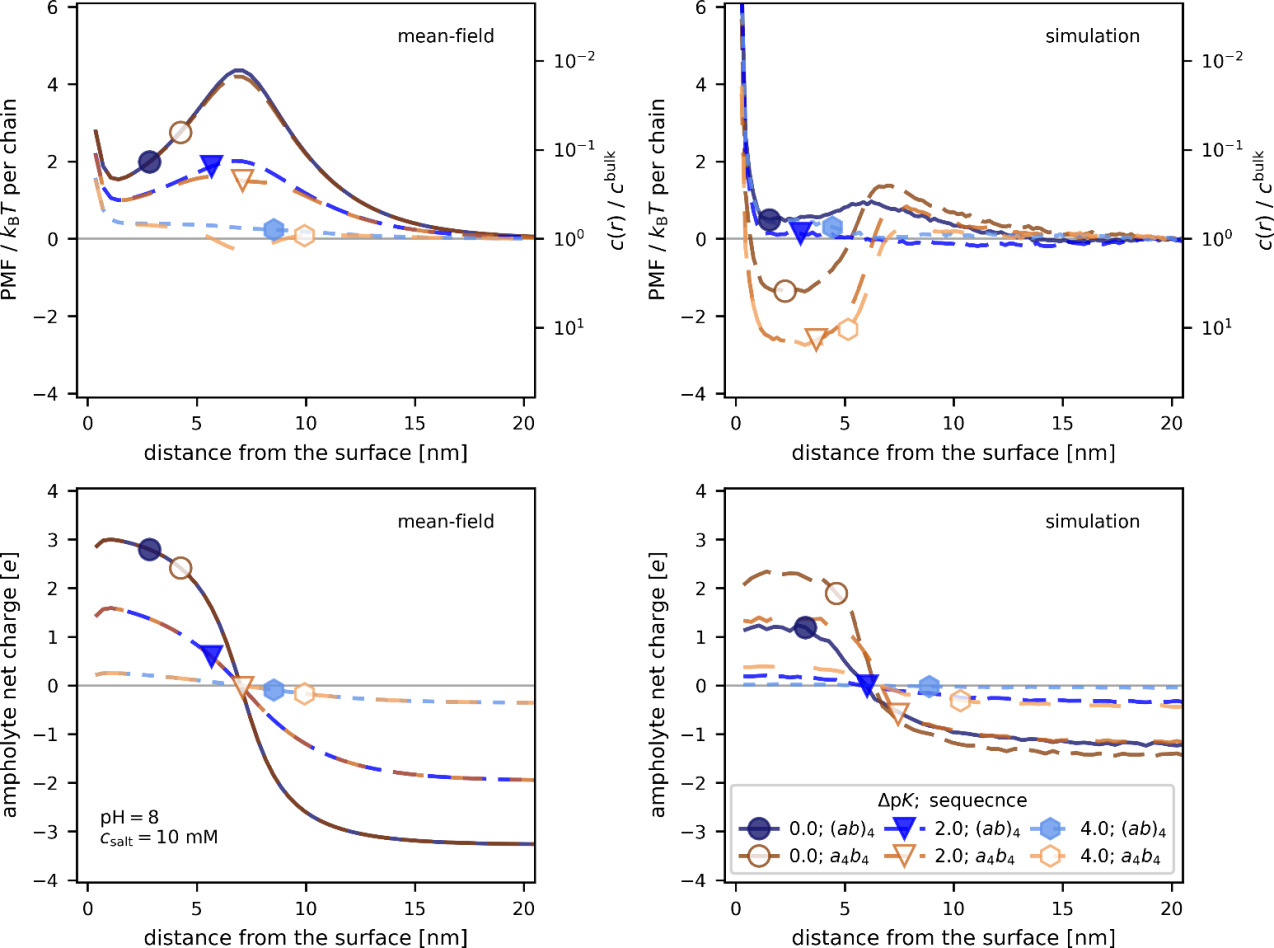
Effect of ampholyte sequence on the PMF, concentration profiles and net charge: alternating sequence (*ab*)_4_ vs block-like patchy sequence *a*_4_ *b*_4_ at pH = 8.0, *c* ^salt^ = 10 mM and Δp*K* ∈ {0, 2, 4}. Potential of mean force (top) and net charge on the ampholyte (bottom). Mean-field results (left) and CG simulations (right).

In summary, we introduced a simple phenomenological model, relating the difference in local pH (electrostatic potential inside and outside the brush) with the titration curve of the ampholyte (dependence of its net charge on the pH). As a proof of concept, we considered a chain-like ampholyte composed of 4 acidic and 4 basic residues, characterized by the difference in their acidity constants, Δp*K*. At pH < pI, the uptake occurs trivially but it can be significantly enhanced by charge regulation. If the difference, δ, in local pH is sufficient to induce a positive net charge in the ampholyte, then we also observe its uptake into the negatively charged brush at pH > pI, triggered by charge regulation. Particularly significant charge regulation occurs if the ampholyte has p*K*_A_ values of acid or base groups close to its isoelectric point. This is in line with earlier observations by other authors, based on numerical mean-field calculations^48,49,51–53,61^ and simulations.^44^ Then, the ampholyte coexists in two very different charge states inside and outside the brush. All of the above effects become more pronounced for ampholytes with a higher number of charge-regulating groups. The same arguments apply to uptake of ampholytic solutes into positively charged brushes, except that the roles of high and low pH are swapped.

Phenomenologically, the extent of the uptake can be estimated from the net charge that the ampholyte would acquire at the local pH inside the brush. The latter can be calculated from the bulk pH in the buffer and Donnan potential across the brush-solution interface. Such an estimate applies not only to the model ampholytes considered here but also to any other ampholytes (peptides, proteins). The only required input is the dependence of their net charge on the pH. By comparing with explicit-particle simulations, we demonstrated that such an estimate works well for alternating sequences of acids and bases, but it fails for block-like (patchy or patterned) sequences. The patchiness generally favors uptake into the brush also in the absence of charge regulation, but it is neglected both in the phenomenological description and mean-field. Our estimate also neglects steric effects and diplolar contributions, which are probably negligible for small molecules but could be significant, especially for bulky proteins. Molecular simulations with explicit particles account for these additional effects, providing more reliable predictions also for more complex solutes. The phenomenological approach presented here can be used as a rule of thumb for predicting the uptake of various charged macromolecules into polymer brushes. However, it is necessary to explicitly account for both charge regulation and charge patterning in order to quantitatively describe the uptake of complex ampholytic solutes, such as longer peptides or proteins, into polyelectrolyte brushes or hydrogels.

## Supplementary Material



## Notes

Technical details of the CG and MF simulation models and methods, density profiles of the brush, PMF and net charge profiles for the (ab)_1_ ampholytes, and PMF profiles for various solute lengths (PDF)
