# Supplementary material for: Charge Regulation Enables Uptake of Ampholytes to Polyelectrolyte Brushes
Source: ACS Macro Lett. 2025 Dec 29;15(1):102–11. doi: 10.1021/acsmacrolett.5c00669 (PMC12825373; doi:10.1021/acsmacrolett.5c00669)
Supplement: Supplementary file 1 [file mz5c00669_si_001.pdf]

Supplementary Material: Charge regulation enables uptake of ampholytes to polyelectrolyte brushes

Roman Staňo[1,*] and Peter Košovan[2,†]

[1] *Yusuf Hamied Department of Chemistry, University of Cambridge, Lensfield Road, Cambridge CB2 1EW, United Kingdom*
[2] *Department of Physical and Macromolecular Chemistry, Faculty of Science, Charles University, Hlavova 8, 128 40 Prague 2, Czech Republic*

(Dated: December 28, 2025)

CONTENTS

I. Simulation Model & Method 1
A. Microscopic model 1
1. System description 1
2. Interaction potentials 2
B. Simulation method 3
1. Langevin dynamics 3
2. Grand-reaction Monte Carlo 3
C. Calculation of potentials of mean force 4

II. Self-consistent Field Model & Method 5
A. The numerical mean-field model 5
B. The Scheutjens-Fleer self-consistent field implementation 5

III. Density profiles of the brush 7

IV. PMF and net charge profiles for $(ab)_1$ peptides 8

V. PMF profiles for various solute lengths 10

References 11

I. SIMULATION MODEL & METHOD

A. Microscopic model

We use a coarse-grained model of a polymer brush, represented as an array of polymer chains grafted to a surface. The brush co-exists with a buffer solution containing a single peptide chain and small ions, all of the above being modelled as explicit particles in implicit solvent. We aim at having a simple generic model with small amount of variables, and we consider only three terms in the potential energy: excluded volume interactions, bonded interactions and electrostatic interactions between the species. The model is not supposed to create a highly accurate representation of a particular experimental system, nevertheless, we choose the interaction parameters within order of magnitude typical for some of the recently explored systems [1].

1. System description

Our system is enclosed in a simulation box, shaped as a square prism with dimensions $L_x = L_y = \sqrt{M/\Gamma}$, where $M = 25$ is the number of polymer chains in the box, Γ is their grafting density, and $L_z = 3L_x$. The system has periodic boundary conditions in dimensions x and y, but not in the z dimension, where the boundaries are modelled

* rs2365@cam.ac.uk
† peter.kosovan@natur.cuni.cz

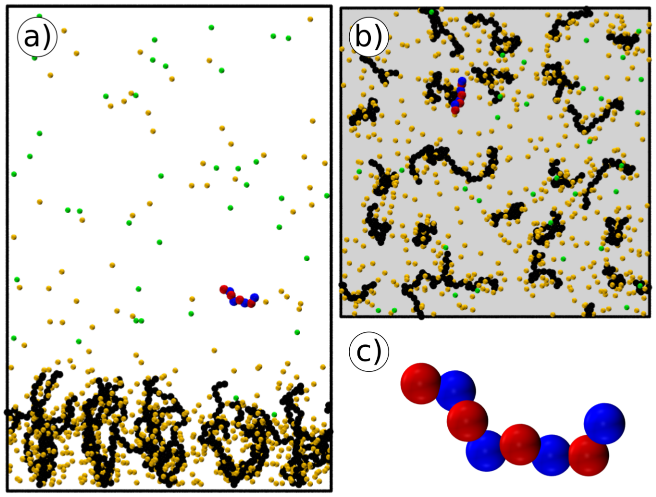

FIG. S1. a) side-view (xz plane) of the simulated system at $\Gamma = 0.079\text{nm}^{-2}$, $c^{\text{salt}} = 0.010$ mol/L and pH = 7.0, $\Delta\text{p}K = 0.0$ and ampholyte $(ab)_4$. b) top-view (xy plane) of the system in the panel a). c) detail of the peptide chain from the panel a).

as hard reflective walls, placed in xy planes at $z = 0$ and at $z = L_z$. As shown before [2], the finite size effects on the conformations of brushes are rather small in boxes of these sizes.

The polymer chains forming the brush are modelled using the standard bead-spring model [3, 4], as homopolymers with monomeric unit denoted here as X^-, carrying a permanent charge of charge number -1 each. Each chain is composed of $N = 25$ monomeric units. The chains are grafted on a primitive square lattice with step $1/\sqrt{\Gamma}$ spanned on the wall at $z = 0$, such that the first monomer of each of the chains is pinned at height $z = \sigma$, its position is constrained and does not evolve in time.

The system contains a single peptide co-polymer chain of length $2n$ and architecture $(ab)_n$ or $a_n b_n$, where a stand for acidic amino-acid and b for a basic amino-acid. The acidic amino-acid is represented by a single particle, which can attain one of two charge states – either negatively charged A^- or neutral HA, the equilibrium being controlled by the acidity constant $\text{p}K_{\text{A}}^{\text{acid}}$ through the Grand-reaction Monte Carlo scheme, as defined later. In a close analogy, the basic amino-acid can attain charge states of BH^+ or B, controlled by the corresponding constant $\text{p}K_{\text{A}}^{\text{base}}$.

Finally, the system contains small ions of type Na^+, Cl^-, H^+ and OH^-, whose counts are not-fixed, but rather fluctuate around their ensemble averages in accordance with the Grand-reaction Monte Carlo scheme, as described later on. The system always carries a net charge of zero.

2. *Interaction potentials*

All of the pairs of particles presented above interact via repulsive Weeks-Chandler-Andersen potential defined as:

$$U_{\text{WCA}}(r) = \begin{cases} 4\epsilon\left[\left(\dfrac{\sigma}{r}\right)^{12} - \left(\dfrac{\sigma}{r}\right)^{6} + \dfrac{1}{4}\right] & r \le 2^{1/6}\sigma, \\ 0 & r > 2^{1/6}\sigma, \end{cases} \tag{S1}$$

where r is the instantaneous distance between the particles, and we set the energy scale by $\epsilon = k_{\text{B}}T$ and the length scale as $\sigma = 0.355$ nm.

All of the bonds between two consecutive monomeric units are emulated by finitely extensible non-linear elastic (FENE) potential given by

$$U_{\text{FENE}}(r) = \begin{cases} -\dfrac{1}{2}K_F R_0^2 \ln\left[1 - \left(\dfrac{r}{R_0}\right)^2\right], & r \le R_0 \\ \infty & r > R_0, \end{cases} \tag{S2}$$

where $K_F = 30 k_{\text{B}}T/\sigma^2$ is the spring constant and $R_0 = 1.5\sigma$ is the maximal extension of the bond, beyond which the potential diverges.

Finally, all of the charged species interact via unscreened long-ranged Coulomb potential

$$U_{\mathrm{C}}(r) = k_{\mathrm{B}} T z_i z_j \frac{l_{\mathrm{B}}}{r}, \tag{S3}$$

where z_i, z_j are the charge numbers and $l_{\mathrm{B}} = e^2/4\pi\varepsilon_{\mathrm{r}}\varepsilon_0 k_{\mathrm{B}} T$ is the Bjerrum length set to $l_{\mathrm{B}} = 2\sigma = 0.710$ nm corresponding to the aqueous solutions at room temperature. The electrostatic interactions are evaluated using the particle-particle particle-mesh (P3M) method [5–7] tuned to the relative accuracy 10^{-3}, together with the electrostatic layer correction (ELC) [8–10] with gap size $\Delta L = 25\sigma$, effectively subtracting the periodic contribution of the P3M method to the forces in the z direction, turning the system into a slab.

B. Simulation method

The simulation method alternates dynamic steps, under which the system evolves using Molecular dynamics, with reaction steps, under which we sample the system composition using Monte Carlo.

1. Langevin dynamics

For the former of the steps, we sample configurations at fixed composition by propagating the particles, indexed i, following the below equations of motion with Langevin coupling

$$m\ddot{\mathbf{x}}_i(t) = \mathbf{F}_i - \gamma m \dot{\mathbf{x}}_i(t) + \mathbf{Y}_i(t), \tag{S4}$$

where γ is the friction coefficient and $\mathbf{Y}$ is a random force such that $\langle \mathbf{Y}_i(t) \rangle = 0$ and $\langle \mathbf{Y}_i(t)\mathbf{Y}_i(t') \rangle = 2\gamma m_i k_{\mathrm{B}} T \delta(t-t')$, where δ is Kronecker delta. The force $\mathbf{F}_i$ is the deterministic force derived from the gradients of the interaction potentials, and for the peptide also the biasing potential described further in the text (Eq. S18). Finally, the mass of each particle is $m = 1$, which sets the time unit as $\tau = (m\sigma^2/k_{\mathrm{B}}T)^{1/2} = 1/\gamma = 1$, and we use a time-step $\Delta t = 0.01\tau$, and the simulations were 10^7 time steps long, which translates to $10^5\tau$ which is several times longer than the time scale at which the arms of the brush retract and relax [11].

2. Grand-reaction Monte Carlo

To simulate the ion exchange and the chemical reaction equilibrium on the peptide, we use the Grand-reaction Monte Carlo method [12]. We consider the following set of (virtual) ion exchange reactions

$$\emptyset \rightleftharpoons \mathrm{Na^+} + \mathrm{Cl^-} \tag{S5}$$
$$\emptyset \rightleftharpoons \mathrm{H^+} + \mathrm{Cl^-} \tag{S6}$$
$$\emptyset \rightleftharpoons \mathrm{Na^+} + \mathrm{OH^-} \tag{S7}$$
$$\emptyset \rightleftharpoons \mathrm{H^+} + \mathrm{OH^-}, \tag{S8}$$

ionization reactions for the acidic sites of the peptide

$$\mathrm{HA} \rightleftharpoons \mathrm{A^-} + \mathrm{H^+} \tag{S9}$$
$$\mathrm{HA} \rightleftharpoons \mathrm{A^-} + \mathrm{Na^+} \tag{S10}$$
$$\mathrm{HA} + \mathrm{Cl^-} \rightleftharpoons \mathrm{A^-} \tag{S11}$$
$$\mathrm{HA} + \mathrm{OH^-} \rightleftharpoons \mathrm{A^-}, \tag{S12}$$

and ionization reactions for the basic sites of the peptide

$$\mathrm{BH^+} \rightleftharpoons \mathrm{B} + \mathrm{H^+} \tag{S13}$$
$$\mathrm{BH^+} \rightleftharpoons \mathrm{B} + \mathrm{Na^+} \tag{S14}$$
$$\mathrm{BH^+} + \mathrm{Cl^-} \rightleftharpoons \mathrm{B} \tag{S15}$$
$$\mathrm{BH^+} + \mathrm{OH^-} \rightleftharpoons \mathrm{B}. \tag{S16}$$

In a single reaction step, we randomly select a reaction among the above sets and the direction of the reaction, both with uniform probabilities. For the exchange reactions, we either randomly insert new particles or remove randomly chosen particles, following the stoichiometry of the reaction chosen to be sampled. For the ionization reactions, random insertion or deletion is applied to the counterion or co-ion, while the acidic or basic site on the peptide just changes the sign, again following the stoichiometry. The result of this manipulation of particles is a trial configuration, which is accepted with the Monte Carlo criterion

$$P_{\mathrm{n,o}}^{\mathrm{G-RxMC}} = \min\left\{1, \left(\prod_i \frac{N_i^0!\,(Vc^{\ominus})^{\nu_i \xi}}{(N_i^0+\nu_i\xi)!}\right) \exp\left(\beta\left[\xi\sum_i \nu_i(\mu_i - \mu_i^{\ominus}) - \Delta\mathcal{U}_{\mathrm{n,o}}\right]\right)\right\}, \tag{S17}$$

where μ_i and $\mu_i^{\ominus}$ are the total chemical potential and the reference chemical potential of the particle i, V is the volume of the system, N_i^0 is the initial number of particles i in the system, $c^{\ominus} = 1\mathrm{mol/L}$ is the standard reference concentration, $\xi = \sum_i \nu_i$ is the extent of the reaction, where ν_i is the stoichiometric coefficient given in the sampled reaction and finally $\mathcal{U}_{\mathrm{n,o}}$ is the potential energy difference between the original configuration and the trial one. Should the trial configuration be rejected, we keep the original configuration.

We attempt 10^3 reaction steps every for every 500 integration steps (5τ), amounting to $2\cdot 10^7$ of the reaction steps over the course of the full simulation.

C. Calculation of potentials of mean force

To improve the sampling of density profiles of peptide in the brush, we use biasing via umbrella sampling method [13–15]. We apply an external potential

$$U_{\mathrm{B}}(z) = \frac{K_{\mathrm{B}}}{2}(z-z_0)^2, \tag{S18}$$

on the z-coordinate of the center of mass of the peptide, where $K_{\mathrm{B}} = 2.5k_{\mathrm{B}}T/\sigma^2$. We carry out a series of 60 independent simulations for $z_0/\sigma \in \{1.0, 2.0, \ldots, 60.0\}$, and the bias is subsequently removed using weighed histogram analysis method (WHAM), iteratively solving the following set of equations to the self-consistency [16]

$$c_{ij} = \exp(-\beta U_{\mathrm{B}}^i(z_j)) \tag{S19}$$

$$f_i^{-1} = \sum_j c_{ij} p_j^{\circ} \tag{S20}$$

$$p_j^{\circ} = \frac{\sum_i^S n_{ij}}{\sum_i N_i f_i c_{ij}}. \tag{S21}$$

Our sought-for result is the discrete probability distribution p_j° of the reaction coordinate z in the interval $[0, 60\sigma]$ with bin size 0.5σ, and the j denotes the index of the bin. In Eq. S21, N_i corresponds to the total number of samples (of the reaction coordinate) collected in the simulation i out of 60 simulations mentioned above, n_{ij} is the count of samples from the simulation i falling within the bin j of the histogram and c_{ij} corresponds to the Boltzmann factor of the bias in this very bin. Finally, Eq. S20 follows from the normalization constraint $\sum_j p_{ij} = 1$. We iteratively solve the equations until the obtained probability distribution changes with factor less than $(1 \ \pm 10^{-4})$ of its value in between the iteration steps.

We then define the potential of mean force of the peptide as $F(z) = -k_{\mathrm{B}}T\ln(p(z)/p_{\mathrm{ideal}}) + C$, where $p(z) = p_j^{\circ}$ and C is an additive constant arbitrarily chosen such that $F(z \to \infty) \approx 0$ for each of the studied systems, since $p_{\mathrm{ideal}}(z)$ is constant.

II. SELF-CONSISTENT FIELD MODEL & METHOD

A. The numerical mean-field model

Our mean-field model of a polyelectrolyte (PE) brush is derived from similar models of star-like PEs [17], based on the Scheutjens-Fleer self-consistent field (SF-SCF) approach described in more detail in Section II B. The SF-SCF formulation is a discrete analogue of the continuous Edwards diffusion equation [18, 19]. The discrete version implemented on a set of planar layers (”the lattice”) with lattice spacing a allows for the full enumeration of all the chain configurations. The brush is assumed to be composed of polymer chains attached to a flat surface. We assume that the system is homogeneous in the xy plane parallel to the grafting surface. In the z direction, perperndicular to the grafting surface, we explicitly account for the radial gradients in the density distributions of all species and the corresponding effective potentials.

We construct the system such that the zeroth layer of the lattice ($z = 0$) is filled with neutral segments representing the surface. The first segments of the polyelectrolytes are grafted to the first layer ($z = 1$), whereas the densities of other segments and free ions are determined by solving the mean-field equations described below. The system size is chosen as $L = 200$ layers, which ensures that the density profiles of all species have converged to their bulk values. The convergence was further verified by running a set of calculations for seleted parameters using $L = 100$ and $L = 300$ and observing that a change in L has negligible impact on all results. The boundary condition on the other side, opposite to the brush, is set by adding one more layer ($z = 201$), where the densities of all mobile species are fixed to their bulk values. In addition to the polymer brush, we introduce salt ions (Na^+, Cl^-) and water. The bulk concentration of salt ions, and of the H^+ ions are fixed in the outermost layer of the box, which defines the boundary condition for the semi-grandcanonical calculation. The concentration of OH^- ions is determined by explicitly considering the autoprotolysis reaction of water. The volume fraction of the solvent (water) is determined from the incompressibility constraint, which requires that the sum of volume fractions of all species is unity at each lattice site. The bulk concentration of the ampholyte is chosen to an arbitrarily 10^{-5}M, which ensures that even if there is a strong uptake into the brush, the presence of the ampholyte does not singificantly affect the brush properties.

B. The Scheutjens-Fleer self-consistent field implementation

In the Scheutjens-Fleer self-consistent field method, SF-SCF, [20, 21] the free energy is expressed as a functional of the density profiles of all components in the system components, i.e. polymer segments, free ions, and the solvent. This method has been described many times in literature. Therefore, we only briefly describe its essential features and refer the reader to the existing literature for further details [17, 21–23]. Within the planar approximation, the system is represented as a set of parallel layers separated by a constant spacing $a = 0.35$nm, matching the segment size used in the CG simulations. The volume of the effective lattice site, a^3, is then used to convert the volume fractions obtained from the SF-SCF calculations to molar concentrations. The one-dimensional planar approximation employed here assumes that the density is constant within each layer (xy plane), thereby reducing the three-dimensional problem to a one-dimensional problem of determining the density profiles which depend only on the z coordinate, perpendicular to the grafting surface. This greatly reduces the number of iteration variables and speeds up the search for the optimum solution.

The mean-field potential $u_x(z)$, experienced by segments of type x in layer z can be expressed as

$$u_x(z) = u'_x(z) + \nu_x e\psi(z) + k_{\mathrm{B}}T \sum_y \chi\langle\varphi_y(z)\rangle\,, \tag{S22}$$

where e is the elementary charge, ν_x the valency of segment x, $\varphi_y(z)$ is the volume fraction of segments of type y, and $\psi(z)$ is the local electrostatic potential. The summation runs over all components y of the system. The first term in (S22) is the Lagrange field due to the incompressibility constraint [21], the second term accounts for the electrostatic interactions, and the last term accounts for the short-ranged interactions by means of the Flory-Huggins parameter χ. The notation $\langle\varphi(z)\rangle$ stands for the density averaged over layers adjacent to z. To represent the athermal solvent conditions for the brush, we used $\chi = 0$ for all pair interactions. Note that this value does not imply that segment-segment interactions are neglected, because the steric repulsion enters the equation via the incompressibility constraint.

The local electrostatic potential, $\psi(z)$, is obtained by solving the discretized Poisson equation in the planar geometry, with the density profiles of all charged species as input parameters. All components in the system follow the Boltzmann distribution with respect to the potentials, therefore the statistical weight of finding a component x in

layer z is given by

$$G_x(z) = \exp(-u_x(z)) \tag{S23}$$

The SF-SCF formulation introduces a propagator scheme, which allows to compute the density profiles from the given potentials, taking into account the connectivity of chain-like molecules and all their possible conformations which satisfy the given constraints – in our case it is fixation of the first segment at $z^* = 1$. Then the propagator scheme is as follows:

$$G_x(z, s|z^*, 1)) = G_x(z) \langle G_x(z, s-1|z^*, 1) \rangle \tag{S24}$$

$$G_x(z, s|N)) = G_x(z) \langle G_x(z, s+1|N) \rangle \tag{S25}$$

where $G_x(z, s|z^*, 1)$ denotes the statistical weight of a segment with ranking number s in layer z for a conformation with segment 1 in layer z^*, and $G_x(z, s|N))$ is the statistical weight of segment s in layer z for a conformation which starts with segment N in any layer. With the initial conditions $G_x(z, N|N) = G_x(z)$ and $G_x(z^*, 1|z^*, 1) = G_x(z^*)$ Eq. S24 and Eq. S25 can be solved recursively. The volume fraction profiles, $\varphi_x(z, s)$, then follow as

$$\varphi_x(z, s) = C_x \frac{G_x(z, s|z^*, 1))G_x(z, s|N))}{G_x(z)} \tag{S26}$$

where C_x is the normalization constant. The volume fraction of each species is related to its molar concentration as $c = \varphi/(1000 N_\mathrm{A} a^3)\,\mathrm{mol/dm}^3$.

For weak acids and bases, it is necessary to consider different states of the ionizable species (polymer segments and water molecules). Then the above equations are modified such that the fraction of species of type j in state k is given by [24]

$$\alpha_{j,k}(z) = \alpha^b_{j,k}(z) G_{j,k}(z)/G_j(z) \tag{S27}$$

where $\alpha^b_{j,k}(z)$ is the corresponding fraction of species j in state k in the bulk, *i.e.*, far from the brush, in the absence of any field. Because the ionized and non-ionized states have different charge, they experience different potentials and attain different statistical weights in each layer. At the same time, the local concentrations of ionized and non-ionized polymer segments are coupled by the relation

$$\frac{\alpha(z)}{1-\alpha(z)} = \frac{K_\mathrm{A}}{c_{\mathrm{H}^+}(z)} \tag{S28}$$

where $\alpha(z)$ is the local degree of ionization in layer z. Similarly, the autoprotolysis of water, determined by its ionic product, $K_\mathrm{w} = 10^{-14}\mathrm{mol}^2\mathrm{dm}^{-6}$, is considered locally within each layer:

$$c_{\mathrm{H}^+}(z)\, c_{\mathrm{OH}^-}(z) = K_\mathrm{w} \tag{S29}$$

In this way, the local variations of c_{H^+} and of the ionization states of the ampholytes are explicitly included in the mean-field model. [24, 25]

The solution of the set of equations starts from an initial guess of the density profiles, which yield the respective potentials and the statistical weights. Application of the propagator scheme provides a new set of density profiles. The self-consistent solution is reached when the old and the new volume fractions match within a pre-defined accuracy threshold. The final set of density profiles and potentials yield an accurate prediction of the free energy within the mean-field approximation, which consists in replacing explicit inter-particle interactions with a mean-field potential in Eq. S22. Due to the variational principle, the mean-field free energy is an upper bound to the true free energy with all correlations included. This difference is inherent to the involved approximation and is not related to the above mentioned accuracy threshold.

III. DENSITY PROFILES OF THE BRUSH

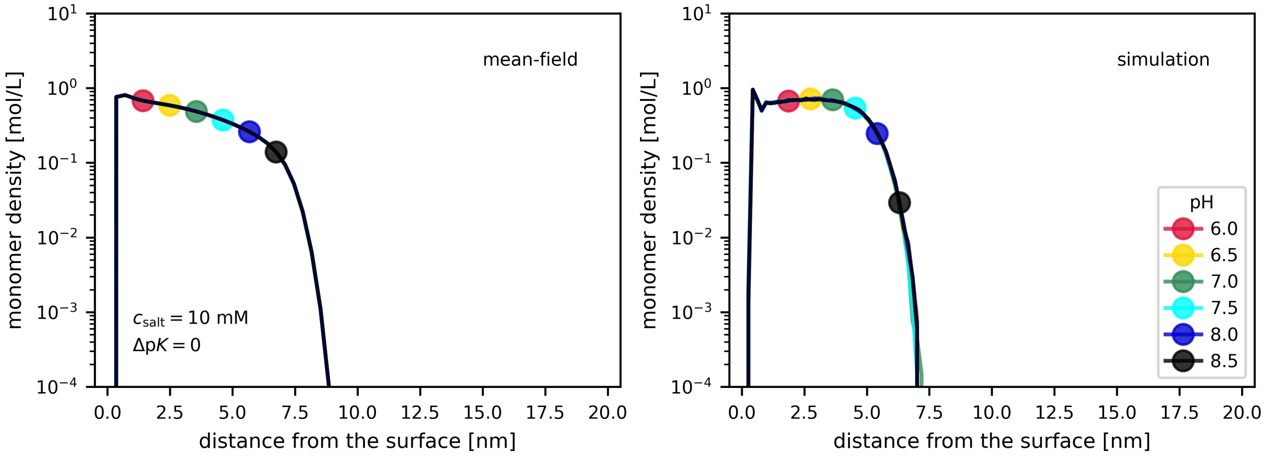

FIG. S2. Density profiles of polymer segments in the brush from the mean-field model (left panel) and CG simulation (right panel). In both cases, the density profiles are practically unaffected by the pH and by the uptake of the ampholyte $(ab)_1$.

IV. PMF AND NET CHARGE PROFILES FOR (ab)$_1$ PEPTIDES

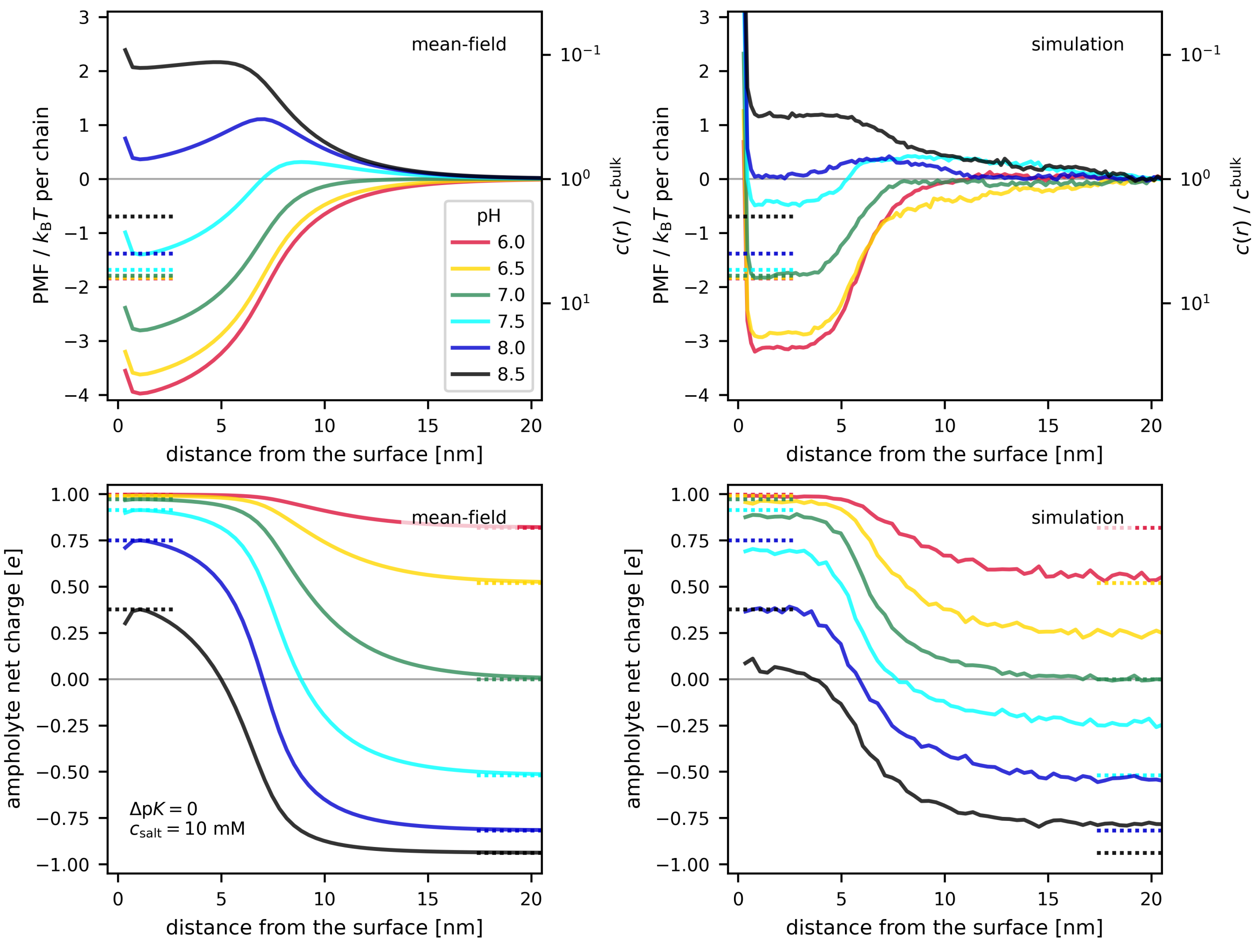

FIG. S3. Uptake of an $(ab)_1$ ampholyte with $\mathrm{p}K_{\mathrm{A}}^{\mathrm{acid}} = \mathrm{p}K_{\mathrm{A}}^{\mathrm{base}} = 7$ in anionic polyelectrolyte brush at various pH values at $c^{\mathrm{salt}} = 10\,\mathrm{mM}$. Dotted horizontal lines are the solution of the two-state phenomenological model. Top row: potentials of mean force (left axis) and local concentration profile (right axis) as a function of distance form the surface; Bottom row: Net charge on the ampholyte as a function of distance form the surface; Left column: results of mean-field calculations; Right column: results of coarse-grained simulations.

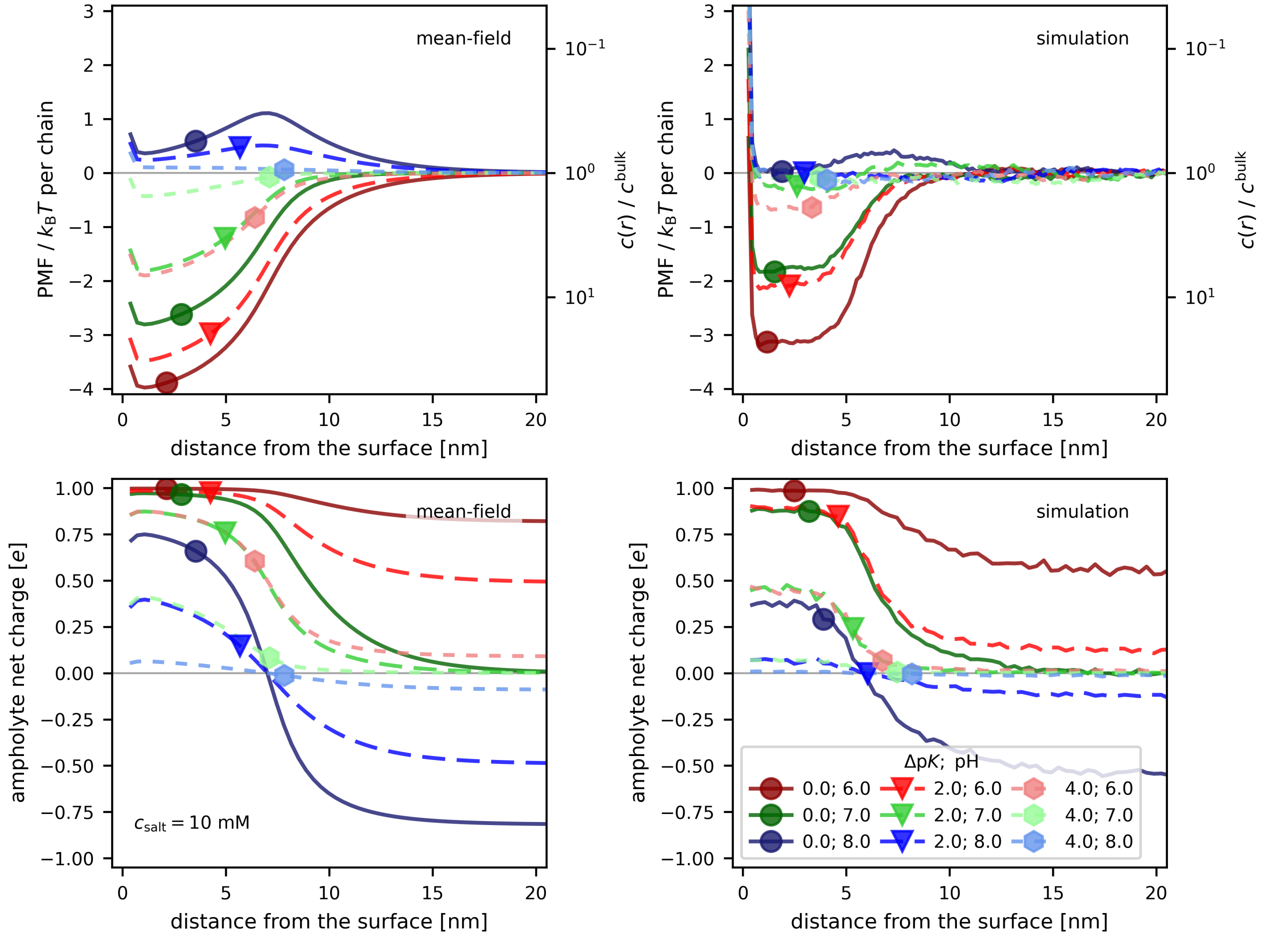

FIG. S4. Uptake of $(ab)_1$ ampholyte with various $\Delta\mathrm{p}K$ values at selected pH values and $c^{\mathrm{salt}} = 10\,\mathrm{mM}$. Top row: potentials of mean force (left axis) and local concentration profile (right axis) as a function of distance form the surface; Bottom row: Net charge on the ampholyte as a function of distance form the surface; Left column: results of mean-field calculations; Right column: results of coarse-grained simulations. Note that some results practically coincide, causing that various types of points appear on the same line.

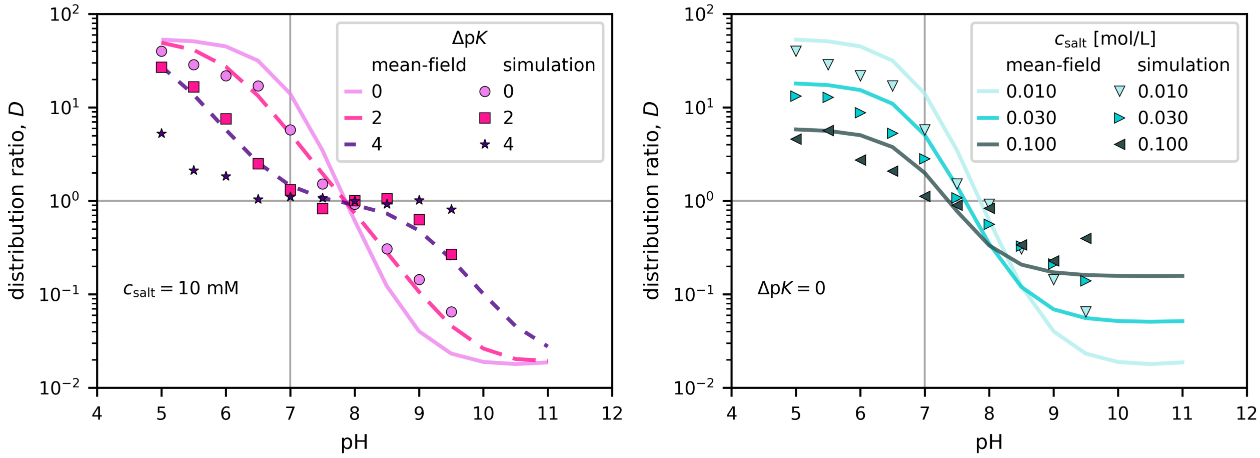

FIG. S5. Distribution ratios of $(ab)_1$ ampholytes between the brush and the bulk as a function of pH under various conditions, comparing mean-field results (lines) with explicit-particle simulations (symbols). Left panel: various values of $\Delta\mathrm{p}K$ at fixed $c^{\mathrm{salt}} = 10\,\mathrm{mM}$. Right panel: various values of c^{salt} at fixed $\Delta\mathrm{p}K = 0$.

V. PMF PROFILES FOR VARIOUS SOLUTE LENGTHS

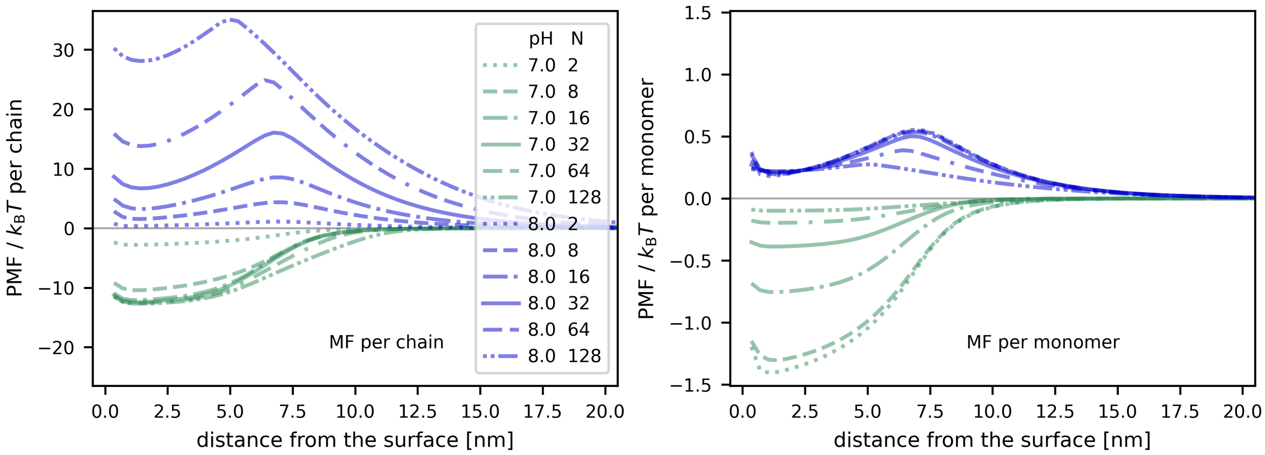

FIG. S6. PMF profiles for alternating solutes with $\Delta pK = 0$ at various number of monomers, N, per solute. Left panel: PMF per chain; Right panel: same data, presented as PMF per monomer.

The low computational cost of the mean-field model allows us to examine how the PMF scales with chain length of the solute. Similar analysis would be very costly in explicit-particle simulations.

For consistency with the rest of the manuscript, we used $N = 25$ monomers per chain in the brush. Therefore, the observed anomalous behaviour of longer solutes could be additionally affected by finite-size effects, as the length of the solute is greater than the length of the brush chains. In principle, it would be possible to use longer chains in the mean-field model to analyze this effect in more detail. Such analysis is presumably beyond the scope of this Letter, as it does not significantly affect the observed phenomena.

[1] G. Ferrand-Drake del Castillo, R. L. N. Hailes, and A. Dahlin, Large changes in protonation of weak polyelectrolyte brushes with salt concentration—implications for protein immobilization, The Journal of Physical Chemistry Letters **11**, 5212–5218 (2020).
[2] D. Beyer, P. Košovan, and C. Holm, Explaining giant apparent pK_a shifts in weak polyelectrolyte brushes, Phys. Rev. Lett. **131**, 168101 (2023).
[3] G. S. Grest and K. Kremer, Molecular dynamics simulation for polymers in the presence of a heat bath, Phys. Rev. A **33**, 3628 (1986).
[4] K. Kremer and G. S. Grest, Dynamics of entangled linear polymer melts: A molecular-dynamics simulation, The Journal of Chemical Physics **92**, 5057 (1990).
[5] R. W. Hockney and J. W. Eastwood, *Computer simulation using particles* (Taylor & Francis, New York, 1988).
[6] M. Deserno and C. Holm, How to mesh up Ewald sums. I. A theoretical and numerical comparison of various particle mesh routines, The Journal of Chemical Physics **109**, 7678 (1998).
[7] M. Deserno and C. Holm, How to mesh up Ewald sums. II. An accurate error estimate for the Particle-Particle-Particle-Mesh algorithm, The Journal of Chemical Physics **109**, 7694 (1998).
[8] A. Arnold, J. de Joannis, and C. Holm, Electrostatics in periodic slab geometries. I, The Journal of Chemical Physics **117**, 2496 (2002).
[9] J. de Joannis, A. Arnold, and C. Holm, Electrostatics in periodic slab geometries. II, The Journal of Chemical Physics **117**, 2503 (2002).
[10] S. Tyagi, A. Arnold, and C. Holm, Electrostatic layer correction with image charges: A linear scaling method to treat slab 2D+h systems with dielectric interfaces, The Journal of Chemical Physics **129**, 204102 (2008).
[11] M. Lang, M. Werner, R. Dockhorn, and T. Kreer, Arm retraction dynamics in dense polymer brushes, Macromolecules **49**, 5190–5201 (2016).
[12] J. Landsgesell, P. Hebbeker, O. Rud, R. Lunkad, P. Košovan, and C. Holm, Grand-reaction method for simulations of ionization equilibria coupled to ion partitioning, Macromolecules **53**, 3007 (2020).
[13] A. M. Ferrenberg and R. H. Swendsen, Optimized monte carlo data analysis, Phys. Rev. Lett. **63**, 1195 (1989).
[14] S. Kumar, J. M. Rosenberg, D. Bouzida, R. H. Swendsen, and P. A. Kollman, The weighted histogram analysis method for free-energy calculations on biomolecules. i. the method, J. Comput. Chem. **13**, 1011 (1992).
[15] D. Frenkel and B. Smit, *Understanding Molecular Simulation: From Algorithms to Applications* (Academic Press, 2002).
[16] M. Andrec, The Weighted Histogram Analysis Method (WHAM) (2010), [Online; accessed 24-August-2022].
[17] J. Klein Wolterink, J. van Male, M. A. Cohen Stuart, L. K. Koopal, E. B. Zhulina, and O. V. Borisov, Annealed star-branched polyelectrolytes in solution, Macromolecules **35**, 9176 (2002).
[18] M. Doi and S. F. Edwards, *The theory of polymer dynamics* (Oxford Science Publications, 1986).
[19] P. G. de Gennes, *Scaling Concepts in Polymer Physics* (Cornell University Press, Ithaca, 1979).
[20] J. Scheutjens and G. Fleer, Statistical-theory of the adsorption of interacting chain molecules 1. partition-function, segment density distribution, and adsorption-isotherms, Journal of Physical Chemistry **83**, 1619 (1979).
[21] G. J. Fleer, M. A. Cohen Stuart, T. Cosgrove, and B. Vincent, *Polymers at interfaces* (Chapman and Hall, London, 1993).
[22] F. Uhlík, P. Košovan, Z. Limpouchová, K. Procházka, O. V. Borisov, and F. A. M. Leermakers, Modeling of ionization and conformations of starlike weak polyelectrolytes, Macromolecules **47**, 4004 (2014).
[23] O. Rud, T. Richter, O. Borisov, C. Holm, and P. Košovan, A self-consistent mean-field model for polyelectrolyte gels, Soft Matter **13**, 3264 (2017).
[24] R. Israëls, F. A. M. Leermakers, and G. J. Fleer, On the theory of grafted weak polyacids, Macromolecules **27**, 3087 (1994), http://pubs.acs.org/doi/pdf/10.1021/ma00089a028.
[25] R. Israëls, F. A. M. Leermakers, G. J. Fleer, and E. B. Zhulina, Charged polymeric brushes: Structure and scaling relations, Macromolecules **27**, 3249 (1994), http://pubs.acs.org/doi/pdf/10.1021/ma00090a018.
